# Multiple genotypes of Crimean-Congo hemorrhagic fever virus detected in ticks during a one health survey in Agnam, Northeastern Senegal

**DOI:** 10.1080/22221751.2022.2136537

**Published:** 2022-11-09

**Authors:** Moufid Mhamadi, Aminata Badji, Idrissa Dieng, Alioune Gaye, El Hadji Ndiaye, Mignane Ndiaye, Moundhir Mhamadi, Cheikh Talibouya Toure, Aliou Barry, Oumar Ndiaye, Babacar Faye, Fatimata Amadou Ba, Boly Diop, Mamadou Ndiaye, Samba Niang Sagne, Gamou Fall, Cheikh Loucoubar, Hugues Fausther Bovendo, Amadou Alpha Sall, Gary Kobinger, Ousmane Faye, Mawlouth Diallo, Oumar Faye

**Affiliations:** a Virology Department, Institut Pasteur de Dakar, Dakar, Senegal; b Medical Zoology Department, Institut Pasteur de Dakar, Dakar, Senegal; c Epidemiology, Clinical Research and Data Science Department, Institut Pasteur de Dakar, Dakar, Senegal; d Institut Pasteur de Dakar, DIATROPIX, Dakar, Senegal; e Parasitology Department, Université Cheikh Anta Diop de Dakar, Dakar, Senegal; f Microbiology, Immunology and Infectious Pathology Service, Department of Public Health and Environment, EISMV of Dakar, Dakar, Senegal; g Ministry of Health and Social Action, Dakar, Senegal; h Global Urgent and Advanced Research and Development, Batiscan, QC, Canada; i University of Texas Medical Branch, Galveston, TX, USA

**Keywords:** CCHF, ticks, Northeastern Senegal, genotypes (I, II & III), livestock

## Abstract

A Crimean-Congo Hemorrhagic Fever Virus (CCHFV) survey in Agnam (North Senegal) permits the detection of three isolates in ticks. These isolates belong genetically to multiple genotypes (I, II, III) and clustered with strains from Uganda, Sudan, Mauritania, and Senegal. The role of ticks in CCHF emergence and widespread is highlighted.

## The study

Crimean-Congo hemorrhagic fever (CCHF) is a tick-borne viral infection. First reported in 1944 in the Crimea region of the Soviet Union and later in 1956 in the current Democratic Republic of Congo [1], nowadays, the disease was reported in more than 30 countries and infested birds could have facilitated this widespread dispersion [2, 3]. The viral genome has three segments, S (Small), M (Medium), and L (Large) and six genotypes were identified [4]. In west Africa, recurrent CCHF cases have been reported [5, 6]. The evidence of CCHF circulation has been reported in Senegal since 1960 [7]. Tick species belonging to *Hyalomma*, *Rhipicephalus*, and *Amblyomma* genus from Senegal can transmit the disease [2, 3, 8]. Epidemiological and environmental data show that Senegal northern regions are the most exposed [9, 10]. Additionally, the last human case was reported in 2019 in Bokidiawe (northern Senegal) [11].

In absence of a specific treatment and vaccine, tick surveillance is the most valuable way for CCHF assessment. Unfortunately, the few data on CCHF surveillance in ticks in Senegal date from the 1990s [2, 3]. For that purpose, Institut Pasteur de Dakar (IPD) established a One Health site in Agnam (16°00′18″N, 13°41′35″W) in northern Senegal to investigate arboviruses and hemorrhagic fever viruses like CCHFV and to update the data on CCHF necessary for the implementation of veterinary vaccines trial which targeting the vectors or the virus itself in a near future by IPD.

For this purpose, blood was taken from every patient with febrile syndrome in the Agnam health care sentinel site located in the One Health site. Blood and ticks were collected from 173 CCHF selected sheep located at Idite (15°55'09.5″N, 13°43'05.1″W) a site located in Agnam. All the samples were tested for serological (ELISA) and molecular (RT-PCR) evidences for CCHFV. Serological test for human and sheep were performed by using an in-house ELISA assay, searching for CCHF antibodies IgM (MAC-ELISA) and IgG (indirect ELISA). For molecular test, RNA was extracted using the QIAamp RNA Viral Kit (Qiagen) according to manufacturer recommendations. CCHFV detection was performed by using the AgPath-ID One-step RT-PCR kit (Thermofisher) with primers & probe previously described [12]. After testing the 173 sheep, only anti-CCHFV IgG was found in 69 sheep (39.88%). The 104 remaining sheep were blood and ticks sampled every 2 weeks until 56 days then monthly until 1 year. Ticks were morphologically identified, pooled by species, then homogenized manually by using sterile mortar and pestle with 0.5–2 mL ice-cold L15 medium in a class 3 Biological Safety Cabinets. The homogenates were clarified by centrifugation and tested by RT-PCR for CCHFV.

From February 2021 to February 2022, we collected 1317 blood samples from sheep and 335 blood samples from febrile humans. No blood samples were tested positive by RT-PCR but 1 anti-CCHFV IgM was detected in a 10-year-old child. He had contact with animal livestock which put him at risk of CCHF infection. Fortunately, the patient recovered without complications.

A total of 2238 ticks were collected and identified: *Hyalomma impeltatum* was the most predominant species (79.71%), followed by *Rhipicephalus guilhoni* (9.83%), *R. muhsamae* (4.83%), *R. evertsi evertsi* (3.44%), *H. truncatum* (1.65%), *H. marginatum rufipes* (0.54%).

We detected three CCHF isolates by RT-PCR, two of them were from *R. evertsi evertsi* and the third was from *R. guilhoni.* All these CCHFV positive ticks were collected on the day of inclusion (Day 0). Full segment amplification was attempted by using the primers previously described [13] and the Q5 High-Fidelity 2X Master Mix (New England Biolabs) according to the manufacturer. Amplicons were obtained from two isolates only. The first one, labelled REE Agnam was detected from *R. evertesi evertesi* and the second isolate labelled RG Agnam was detected from *R. guilhoni.* Low viral load in sample could explain why the third isolate was not amplified. Amplicons were used for sequencing using Nanopore Sequencing. Briefly, amplicons were tagged using the Rapid Barcoding Kit 96 (SQK-RBK110.96). The obtained libraries were quantified, normalized, pooled and loaded on a R 9.4.1 flow cells (FLO-MIN106D) on the Oxford Nanopore MinION platform. The generated reads were basecalled using guppy and merged to a single Fastq file, then consensus sequences were generated with genome detective (https://www.genomedetective.com/). The S and M segments had a good coverage whilst only 6276 bp of the L segments were good for use. As the sequencing protocol is an amplicon-based sequencing, then mutations could lead to drop out in certain genomic region and could explain why we did not obtain good coverage for other segments. Genetic identity was determined by a nucleotide BLAST. Nucleotides sequences were translated to amino-acid and the ClustalW algorithm was used for sequences alignments in bioedit software [14] by using some available sequences of known genotypes [4]. Maximum likelihood (ML) tree construction was performed in iQ-TREE software [15] on the Tamura 3 evolution model with gamma-distributed rates and 1000 non-parametric bootstrap. Aigai virus (ABB30038, ABB30012, ABB30025) was used as outgroup for S, M, and L segments.

**Figure 1.**
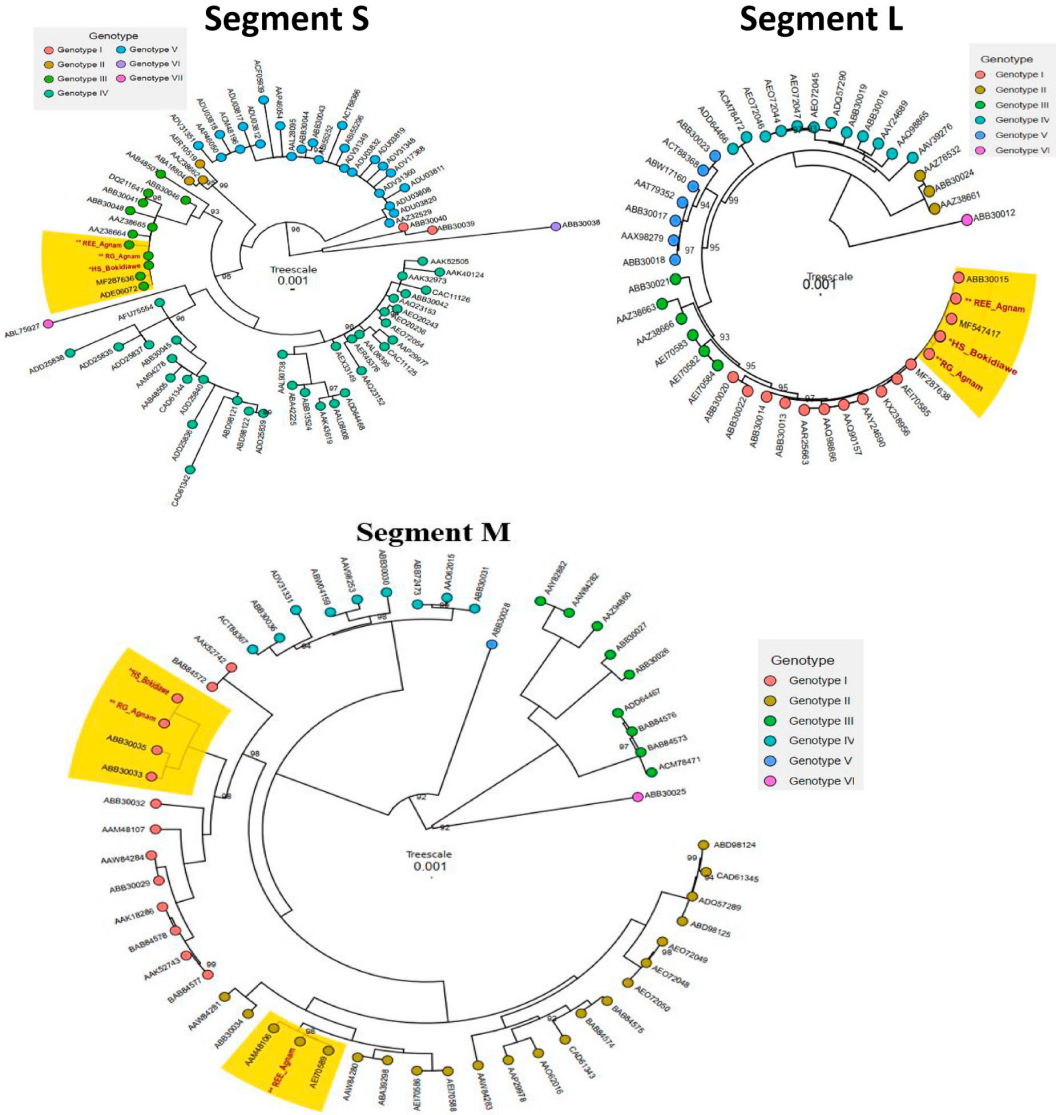
Maximum likelihood trees obtained from the S, M, and L segments analysis based on the genotypes recently updated [4]. All bootstrap values are represented.

Phylogenetic analysis shows that our strains belong to the same genotype (III and I) for the segments L and S where they belong to different genotypes for the segment M respectively genotype I for RG Agnam and genotype II for the REE Agnam. Furthermore, the RG Agnam clustered with the human strain from Bokidiawe (HS Bokidiawe) an area close to Agnam for the 3 segments whereas the REE Agnam seems to have multiple origins because it clustered with the Ugandan (AAZ38664), Sudanese (AEI70589), and Mauritanian (ABB30015) strains for the S, M, and L segments respectively (Figure 1). 99.77%, 97.29%, and 94.83% of amino acid sites were well conserved among HS Bokidiawe, REE Agnam and RG Agnam strains for S, M, and L respectively. These results show that CCHV highly circulates in Agnam and suggest that this area is at high risk for CCHFV emergence by introduction. This result highlighted the usefulness of the CCHFV survey in ticks in the northern Senegal areas to prevent CCHFV emergence, especially since the last human CCHF reported human case in Senegal HS Bokidiawe clustered with the tick isolate RG Agnam SEN 2021 strain. This finding could suggest a virus maintenance of the Bokidiawe strain by ticks and other reservoirs in these two areas and could lead to a CCHFV emergence in the near future. This hypothesis is strengthened by the fact that during the survey on humans, we detected one anti-CCHFV IgM positive in a febrile patient.
